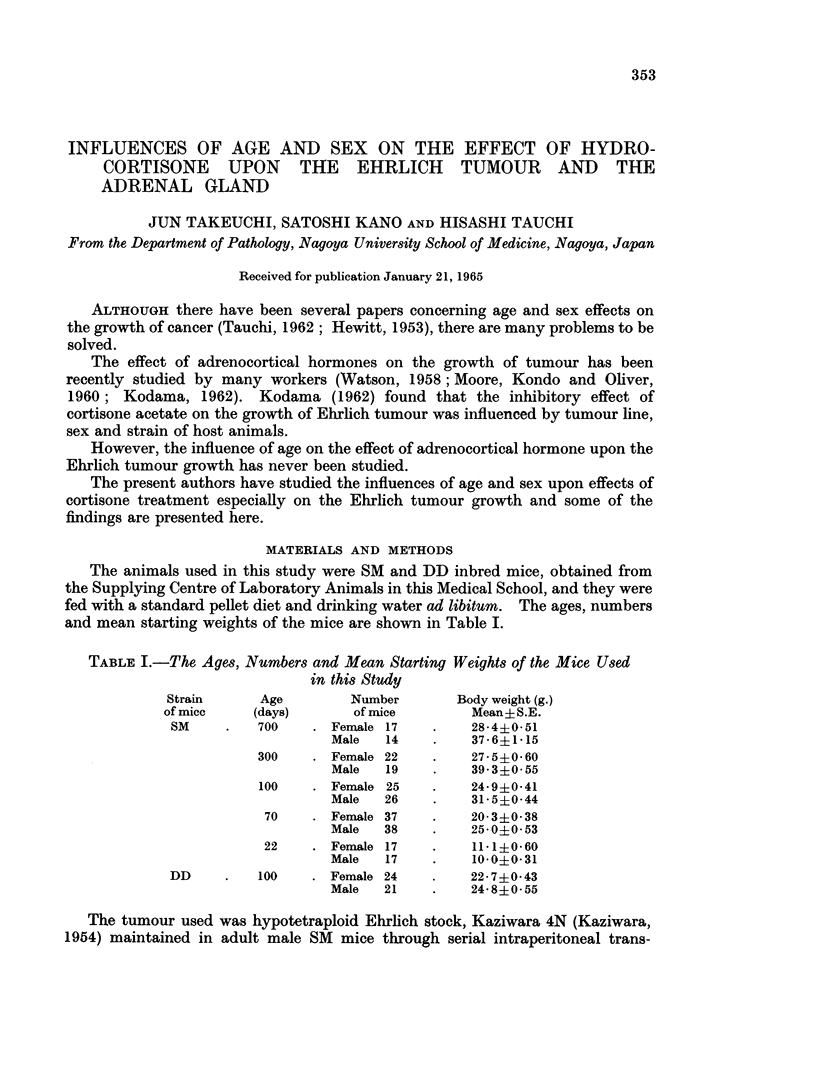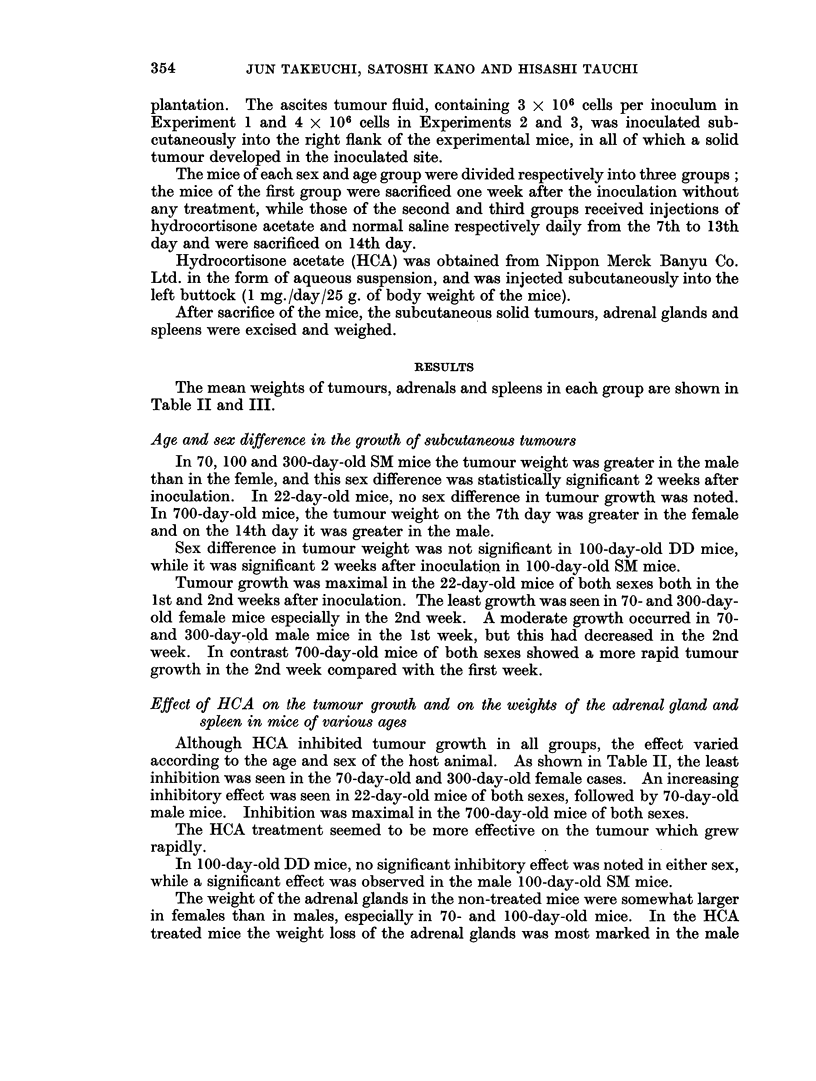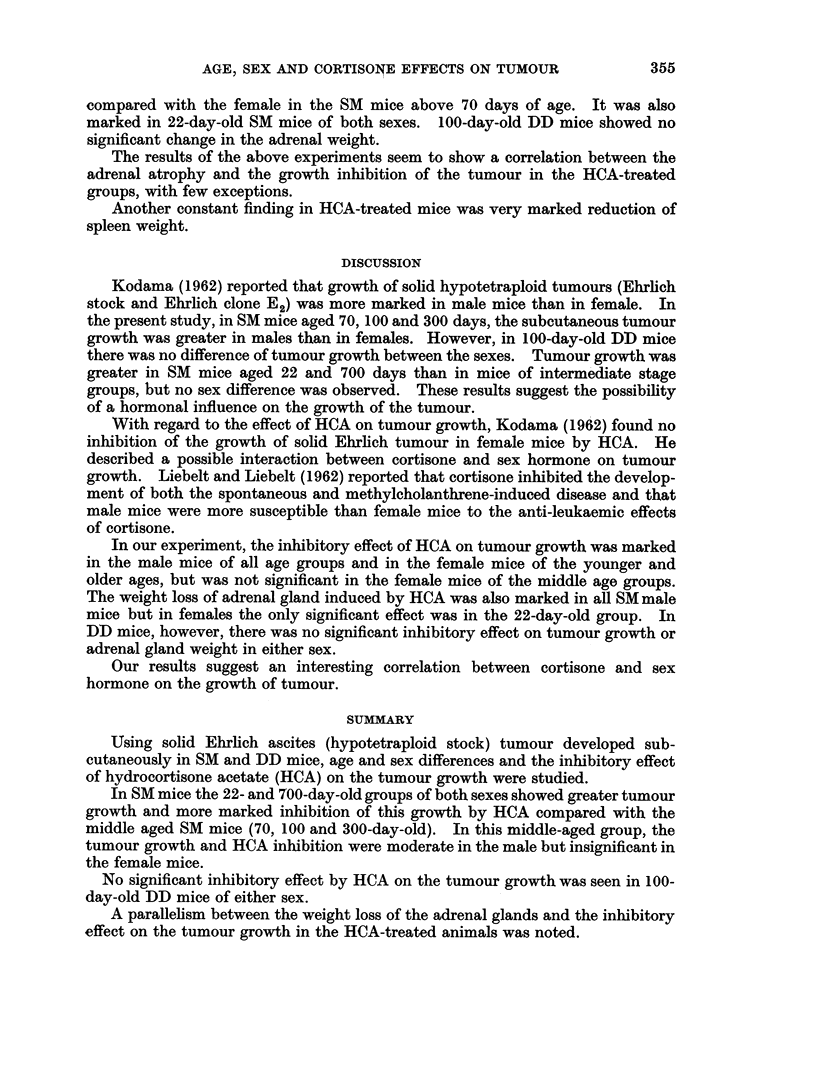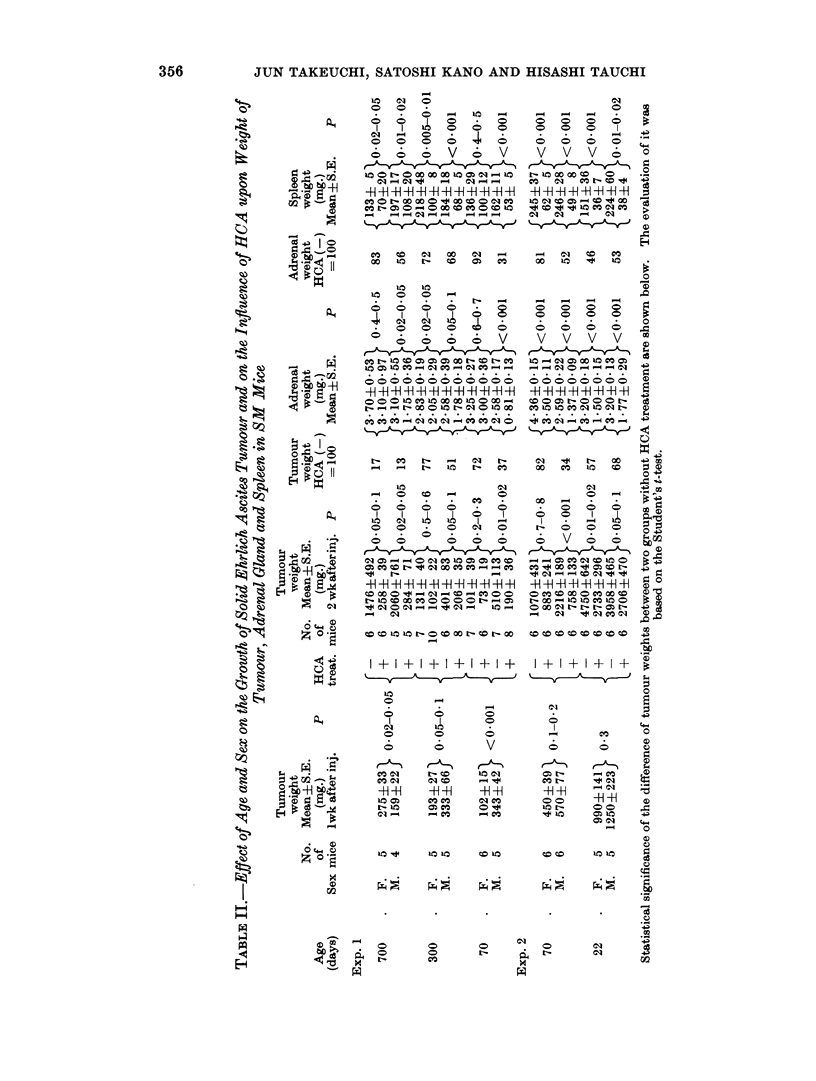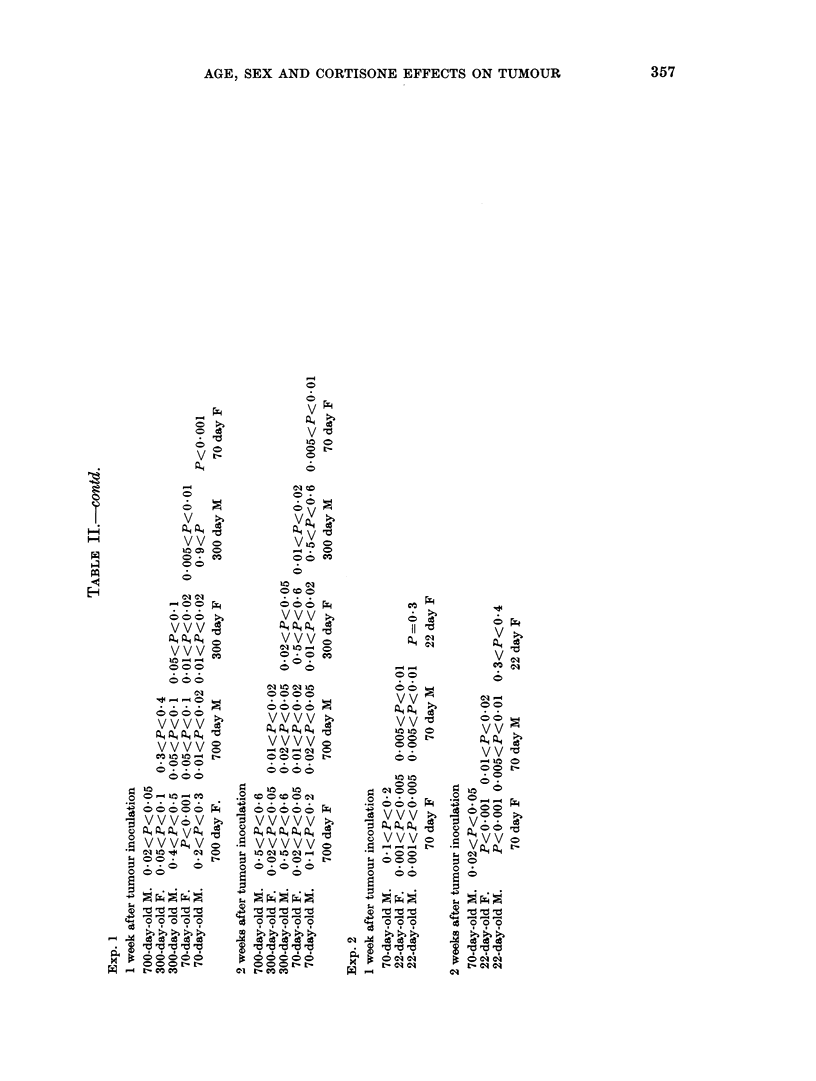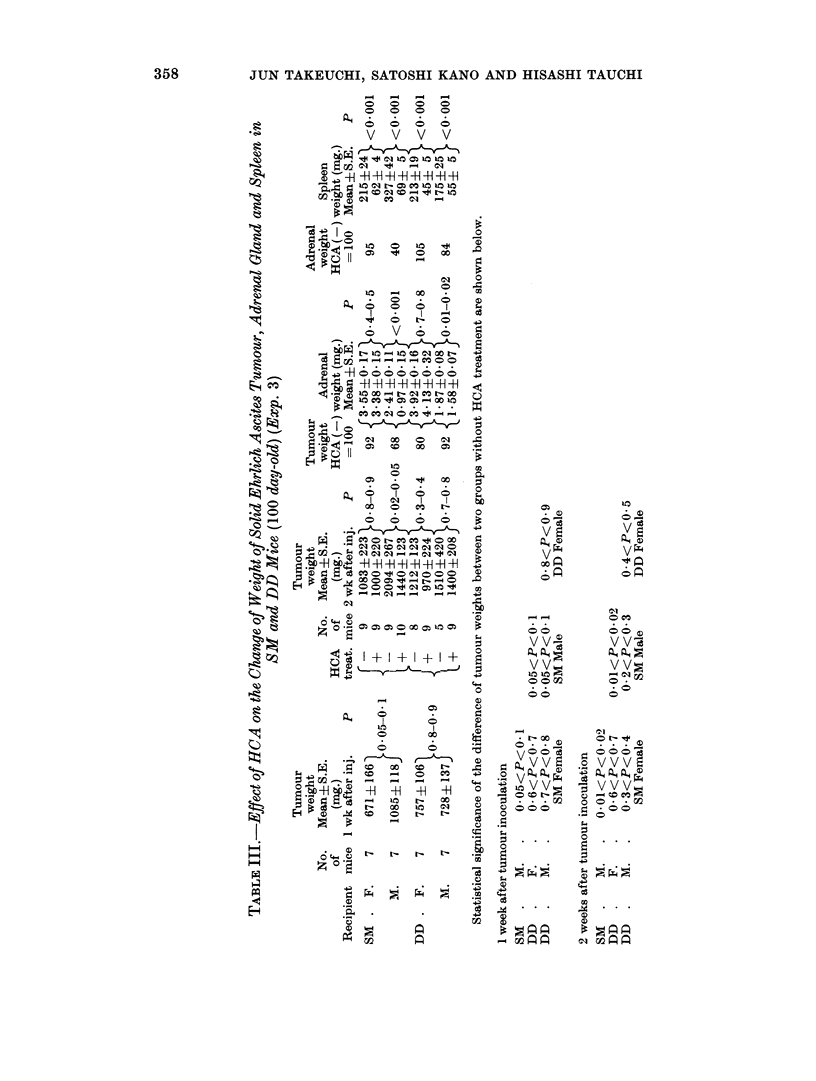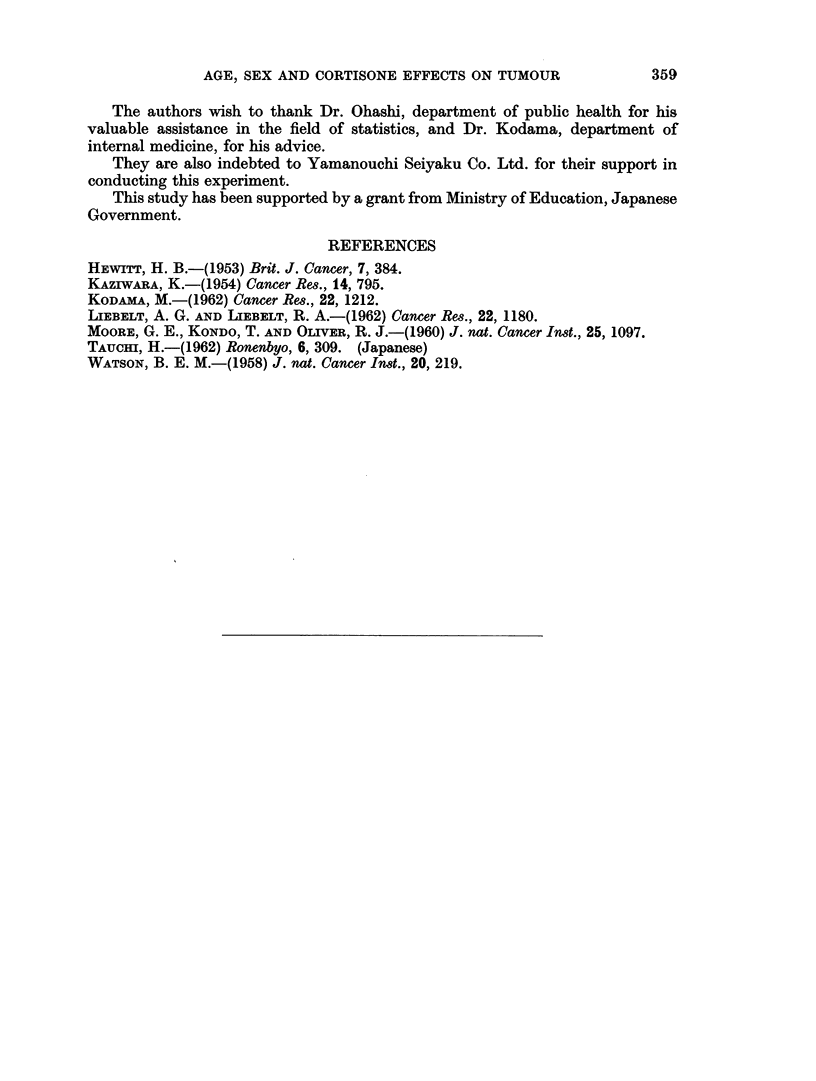# Influences of Age and Sex on the Effect of Hydrocortisone upon the Ehrlich Tumour and the Adrenal Gland

**DOI:** 10.1038/bjc.1965.42

**Published:** 1965-06

**Authors:** Jun Takeuchi, Satoshi Kano, Hisashi Tauchi


### INFLUENCES OF AGE AND SEX ON THE EFFECT OF HYDROCORTISONE UPON THE EHRLICH TUMOUR AND THE ADRENAL GLAND

JUN TAKEUCHI, SATOSHI KANO AND HISASHI TAUCHI
*From the Department of Pathology, Nagoya University School of Medicine, Nagoya, Japan*

Received for publication January 21, 1965

ALTHOUGH there have been several papers concerning age and sex effects on the growth of cancer (Tauchi, 1962 ; Hewitt, 1953), there are many problems to be solved.

The effect of adrenocortical hormones on the growth of tumour has been recently studied by many workers (Watson, 1958 ; Moore, Kondo and Oliver, 1960 ; Kodama, 1962). Kodama (1962) found that the inhibitory effect of cortisone acetate on the growth of Ehrlich tumour was influenced by tumour line, sex and strain of host animals.

However, the influence of age on the effect of adrenocortical hormone upon the Ehrlich tumour growth has never been studied.

The present authors have studied the influences of age and sex upon effects of cortisone treatment especially on the Ehrlich tumour growth and some of the findings are presented here.

### MATERIALS AND METHODS

The animals used in this study were SM and DD inbred mice, obtained from the Supplying Centre of Laboratory Animals in this Medical School, and they were fed with a standard pellet diet and drinking water *ad libitum*. The ages, numbers and mean starting weights of the mice are shown in Table I.

TABLE I.—*The Ages, Numbers and Mean Starting Weights of the Mice Used in this Study*

| Strain of micc | Age (days) | Number of mice | | Body weight (g.) Mean±S.E. |
|---|---|---|---|---|
| SM | 700 | Female | 17 | 28·4±0·51 |
| | | Male | 14 | 37·6±1·15 |
| | 300 | Female | 22 | 27·5±0·60 |
| | | Male | 19 | 39·3±0·55 |
| | 100 | Female | 25 | 24·9±0·41 |
| | | Male | 26 | 31·5±0·44 |
| | 70 | Female | 37 | 20·3±0·38 |
| | | Male | 38 | 25·0±0·53 |
| | 22 | Female | 17 | 11·1±0·60 |
| | | Male | 17 | 10·0±0·31 |
| DD | 100 | Female | 24 | 22·7±0·43 |
| | | Male | 21 | 24·8±0·55 |

The tumour used was hypotetraploid Ehrlich stock, Kaziwara 4N (Kaziwara, 1954) maintained in adult male SM mice through serial intraperitoneal transplantation. The ascites tumour fluid, containing $3 \times 10^6$ cells per inoculum in Experiment 1 and $4 \times 10^6$ cells in Experiments 2 and 3, was inoculated subcutaneously into the right flank of the experimental mice, in all of which a solid tumour developed in the inoculated site.

The mice of each sex and age group were divided respectively into three groups ; the mice of the first group were sacrificed one week after the inoculation without any treatment, while those of the second and third groups received injections of hydrocortisone acetate and normal saline respectively daily from the 7th to 13th day and were sacrificed on 14th day.

Hydrocortisone acetate (HCA) was obtained from Nippon Merck Banyu Co. Ltd. in the form of aqueous suspension, and was injected subcutaneously into the left buttock (1 mg./day/25 g. of body weight of the mice).

After sacrifice of the mice, the subcutaneous solid tumours, adrenal glands and spleens were excised and weighed.

### RESULTS

The mean weights of tumours, adrenals and spleens in each group are shown in Table II and III.

*Age and sex difference in the growth of subcutaneous tumours*

In 70, 100 and 300-day-old SM mice the tumour weight was greater in the male than in the femle, and this sex difference was statistically significant 2 weeks after inoculation. In 22-day-old mice, no sex difference in tumour growth was noted. In 700-day-old mice, the tumour weight on the 7th day was greater in the female and on the 14th day it was greater in the male.

Sex difference in tumour weight was not significant in 100-day-old DD mice, while it was significant 2 weeks after inoculation in 100-day-old SM mice.

Tumour growth was maximal in the 22-day-old mice of both sexes both in the 1st and 2nd weeks after inoculation. The least growth was seen in 70- and 300-day-old female mice especially in the 2nd week. A moderate growth occurred in 70- and 300-day-old male mice in the 1st week, but this had decreased in the 2nd week. In contrast 700-day-old mice of both sexes showed a more rapid tumour growth in the 2nd week compared with the first week.

*Effect of HCA on the tumour growth and on the weights of the adrenal gland and spleen in mice of various ages*

Although HCA inhibited tumour growth in all groups, the effect varied according to the age and sex of the host animal. As shown in Table II, the least inhibition was seen in the 70-day-old and 300-day-old female cases. An increasing inhibitory effect was seen in 22-day-old mice of both sexes, followed by 70-day-old male mice. Inhibition was maximal in the 700-day-old mice of both sexes.

The HCA treatment seemed to be more effective on the tumour which grew rapidly.

In 100-day-old DD mice, no significant inhibitory effect was noted in either sex, while a significant effect was observed in the male 100-day-old SM mice.

The weight of the adrenal glands in the non-treated mice were somewhat larger in females than in males, especially in 70- and 100-day-old mice. In the HCA treated mice the weight loss of the adrenal glands was most marked in the male compared with the female in the SM mice above 70 days of age. It was also marked in 22-day-old SM mice of both sexes. 100-day-old DD mice showed no significant change in the adrenal weight.

The results of the above experiments seem to show a correlation between the adrenal atrophy and the growth inhibition of the tumour in the HCA-treated groups, with few exceptions.

Another constant finding in HCA-treated mice was very marked reduction of spleen weight.

### DISCUSSION

Kodama (1962) reported that growth of solid hypotetraploid tumours (Ehrlich stock and Ehrlich clone $E_2$) was more marked in male mice than in female. In the present study, in SM mice aged 70, 100 and 300 days, the subcutaneous tumour growth was greater in males than in females. However, in 100-day-old DD mice there was no difference of tumour growth between the sexes. Tumour growth was greater in SM mice aged 22 and 700 days than in mice of intermediate stage groups, but no sex difference was observed. These results suggest the possibility of a hormonal influence on the growth of the tumour.

With regard to the effect of HCA on tumour growth, Kodama (1962) found no inhibition of the growth of solid Ehrlich tumour in female mice by HCA. He described a possible interaction between cortisone and sex hormone on tumour growth. Liebelt and Liebelt (1962) reported that cortisone inhibited the development of both the spontaneous and methylcholanthrene-induced disease and that male mice were more susceptible than female mice to the anti-leukaemic effects of cortisone.

In our experiment, the inhibitory effect of HCA on tumour growth was marked in the male mice of all age groups and in the female mice of the younger and older ages, but was not significant in the female mice of the middle age groups. The weight loss of adrenal gland induced by HCA was also marked in all SM male mice but in females the only significant effect was in the 22-day-old group. In DD mice, however, there was no significant inhibitory effect on tumour growth or adrenal gland weight in either sex.

Our results suggest an interesting correlation between cortisone and sex hormone on the growth of tumour.

### SUMMARY

Using solid Ehrlich ascites (hypotetraploid stock) tumour developed subcutaneously in SM and DD mice, age and sex differences and the inhibitory effect of hydrocortisone acetate (HCA) on the tumour growth were studied.

In SM mice the 22- and 700-day-old groups of both sexes showed greater tumour growth and more marked inhibition of this growth by HCA compared with the middle aged SM mice (70, 100 and 300-day-old). In this middle-aged group, the tumour growth and HCA inhibition were moderate in the male but insignificant in the female mice.

No significant inhibitory effect by HCA on the tumour growth was seen in 100-day-old DD mice of either sex.

A parallelism between the weight loss of the adrenal glands and the inhibitory effect on the tumour growth in the HCA-treated animals was noted.

TABLE II.—*Effect of Age and Sex on the Growth of Solid Ehrlich Ascites Tumour and on the Influence of HCA upon Weight of Tumour, Adrenal Gland and Spleen in SM Mice*

| Age (days) | Sex | No. of mice | Tumour weight Mean±S.E. (mg.) 1wk after inj. | *P* | HCA treat. | No. of mice | Tumour weight Mean±S.E. (mg.) 2 wk after inj. | *P* | Tumour weight HCA (−) =100 | Adrenal weight (mg.) Mean±S.E. | *P* | Adrenal weight HCA (−) =100 | Spleen weight (mg.) Mean±S.E. | *P* |
|---|---|---|---|---|---|---|---|---|---|---|---|---|---|---|
| Exp. 1 | | | | | | | | | | | | | | |
| 700 | F. | 5 | 275±33 | 0·02–0·05 | − | 6 | 1476±492 | 0·05–0·1 | 17 | 3·70±0·53 | 0·4–0·5 | 83 | 133± 5 | 0·02–0·05 |
| | | | | | + | 6 | 258± 39 | | | 3·10±0·97 | | | 70±20 | |
| | M. | 4 | 159±22 | | − | 5 | 2060±761 | 0·02–0·05 | 13 | 3·10±0·55 | 0·02–0·05 | 56 | 197±17 | 0·01–0·02 |
| | | | | | + | 5 | 284± 71 | | | 1·75±0·36 | | | 108±20 | |
| 300 | F. | 5 | 193±27 | 0·05–0·1 | − | 7 | 131± 40 | 0·5–0·6 | 77 | 2·83±0·19 | 0·02–0·05 | 72 | 218±48 | 0·005–0·01 |
| | | | | | + | 10 | 102± 22 | | | 2·05±0·29 | | | 100± 8 | |
| | M. | 5 | 333±66 | | − | 6 | 401± 83 | 0·05–0·1 | 51 | 2·58±0·39 | 0·05–0·1 | 68 | 184±18 | <0·001 |
| | | | | | + | 8 | 206± 35 | | | 1·78±0·18 | | | 68± 5 | |
| 70 | F. | 6 | 102±15 | <0·001 | − | 7 | 101± 39 | 0·2–0·3 | 72 | 3·25±0·27 | 0·6–0·7 | 92 | 136±29 | 0·4–0·5 |
| | | | | | + | 6 | 73± 19 | | | 3·00±0·36 | | | 100±12 | |
| | M. | 5 | 343±42 | | − | 7 | 510±113 | 0·01–0·02 | 37 | 2·58±0·17 | <0·001 | 31 | 162±11 | <0·001 |
| | | | | | + | 8 | 190± 36 | | | 0·81±0·13 | | | 53± 5 | |
| Exp. 2 | | | | | | | | | | | | | | |
| 70 | F. | 6 | 450±39 | 0·1–0·2 | − | 6 | 1070±431 | 0·7–0·8 | 82 | 4·36±0·15 | <0·001 | 81 | 245±37 | <0·001 |
| | | | | | + | 6 | 883±241 | | | 3·50±0·11 | | | 62± 5 | |
| | M. | 6 | 570±77 | | − | 6 | 2216±189 | <0·001 | 34 | 2·59±0·22 | <0·001 | 52 | 246±28 | <0·001 |
| | | | | | + | 6 | 758±133 | | | 1·37±0·09 | | | 49± 8 | |
| 22 | F. | 5 | 990±141 | 0·3 | − | 6 | 4750±642 | 0·01–0·02 | 57 | 3·20±0·18 | <0·001 | 46 | 151±36 | <0·001 |
| | | | | | + | 6 | 2733±296 | | | 1·50±0·15 | | | 36±7 | |
| | M. | 5 | 1250±223 | | − | 6 | 3958±465 | 0·05–0·1 | 68 | 3·20±0·13 | <0·001 | 53 | 224±60 | 0·01–0·02 |
| | | | | | + | 6 | 2706±470 | | | 1·77±0·29 | | | 38±4 | |

Statistical significance of the difference of tumour weights between two groups without HCA treatment are shown below. The evaluation of it was based on the Student's *t*-test.

TABLE II.—*contd.*

Exp. 1

1 week after tumour inoculation

| | | | | | |
|---|---|---|---|---|---|
| 700-day-old M. | $0 \cdot 02 < P < 0 \cdot 05$ | | | | |
| 300-day-old F. | $0 \cdot 05 < P < 0 \cdot 1$ | $0 \cdot 3 < P < 0 \cdot 4$ | | | |
| 300-day old M. | $0 \cdot 4 < P < 0 \cdot 5$ | $0 \cdot 05 < P < 0 \cdot 1$ | $0 \cdot 05 < P < 0 \cdot 1$ | | |
| 70-day-old F. | $P < 0 \cdot 001$ | $0 \cdot 05 < P < 0 \cdot 1$ | $0 \cdot 01 < P < 0 \cdot 02$ | $0 \cdot 005 < P < 0 \cdot 01$ | |
| 70-day-old M. | $0 \cdot 2 < P < 0 \cdot 3$ | $0 \cdot 01 < P < 0 \cdot 02$ | $0 \cdot 01 < P < 0 \cdot 02$ | $0 \cdot 9 < P$ | $P < 0 \cdot 001$ |
| | 700 day F. | 700 day M | 300 day F | 300 day M | 70 day F |

2 weeks after tumour inoculation

| | | | | | |
|---|---|---|---|---|---|
| 700-day-old M. | $0 \cdot 5 < P < 0 \cdot 6$ | | | | |
| 300-day-old F. | $0 \cdot 02 < P < 0 \cdot 05$ | $0 \cdot 01 < P < 0 \cdot 02$ | | | |
| 300-day-old M. | $0 \cdot 5 < P < 0 \cdot 6$ | $0 \cdot 02 < P < 0 \cdot 05$ | $0 \cdot 02 < P < 0 \cdot 05$ | | |
| 70-day-old F. | $0 \cdot 02 < P < 0 \cdot 05$ | $0 \cdot 01 < P < 0 \cdot 02$ | $0 \cdot 5 < P < 0 \cdot 6$ | $0 \cdot 01 < P < 0 \cdot 02$ | |
| 70-day-old M. | $0 \cdot 1 < P < 0 \cdot 2$ | $0 \cdot 02 < P < 0 \cdot 05$ | $0 \cdot 01 < P < 0 \cdot 02$ | $0 \cdot 5 < P < 0 \cdot 6$ | $0 \cdot 005 < P < 0 \cdot 01$ |
| | 700 day F | 700 day M | 300 day F | 300 day M | 70 day F |

Exp. 2

1 week after tumour incoulation

| | | | |
|---|---|---|---|
| 70-day-old M. | $0 \cdot 1 < P < 0 \cdot 2$ | | |
| 22-day-old F. | $0 \cdot 001 < P < 0 \cdot 005$ | $0 \cdot 005 < P < 0 \cdot 01$ | |
| 22-day-old M. | $0 \cdot 001 < P < 0 \cdot 005$ | $0 \cdot 005 < P < 0 \cdot 01$ | $P = 0 \cdot 3$ |
| | 70 day F | 70 day M | 22 day F |

2 weeks after tumour inoculation

| | | | |
|---|---|---|---|
| 70-day-old M. | $0 \cdot 02 < P < 0 \cdot 05$ | | |
| 22-day-old F. | $P < 0 \cdot 001$ | $0 \cdot 01 < P < 0 \cdot 02$ | |
| 22-day-old M. | $P < 0 \cdot 001$ | $0 \cdot 005 < P < 0 \cdot 01$ | $0 \cdot 3 < P < 0 \cdot 4$ |
| | 70 day F | 70 day M | 22 day F |

TABLE III.—*Effect of HCA on the Change of Weight of Solid Ehrlich Ascites Tumour, Adrenal Gland and Spleen in SM and DD Mice* (100 *day-old*) (*Exp.* 3)

| Recipient | | No. of mice | Tumour weight Mean±S.E. (mg.) 1 wk after inj. | P | HCA treat. | No. of mice | Tumour weight Mean±S.E. (mg.) 2 wk after inj. | P | Tumour weight HCA (−) =100 | Adrenal weight (mg.) Mean±S.E. | P | Adrenal weight HCA (−) =100 | Spleen weight (mg.) Mean±S.E. | P |
|---|---|---|---|---|---|---|---|---|---|---|---|---|---|---|
| SM . | F. | 7 | 671±166 | 0·05–0·1 | − | 9 | 1083±223 | 0·8–0·9 | 92 | 3·55±0·17 | 0·4–0·5 | 95 | 215±24 | <0·001 |
| | | | | | + | 9 | 1000±220 | | | 3·38±0·15 | | | 62± 4 | |
| | M. | 7 | 1085±118 | | − | 9 | 2094±267 | 0·02–0·05 | 68 | 2·41±0·11 | <0·001 | 40 | 327±42 | <0·001 |
| | | | | | + | 10 | 1440±123 | | | 0·97±0·15 | | | 69± 5 | |
| DD . | F. | 7 | 757±106 | 0·8–0·9 | − | 8 | 1212±123 | 0·3–0·4 | 80 | 3·92±0·16 | 0·7–0·8 | 105 | 213±19 | <0·001 |
| | | | | | + | 9 | 970±224 | | | 4·13±0·32 | | | 45± 5 | |
| | M. | 7 | 728±137 | | − | 5 | 1510±420 | 0·7–0·8 | 92 | 1·87±0·08 | 0·01–0·02 | 84 | 175±25 | <0·001 |
| | | | | | + | 9 | 1400±208 | | | 1·58±0·07 | | | 55± 5 | |

Statistical significance of the difference of tumour weights between two groups without HCA treatment are shown below.

| | | SM Female | SM Male | DD Female |
|---|---|---|---|---|
| 1 week after tumour inoculation | | | | |
| SM . | M. . | $0·05<P<0·1$ | | |
| DD . | F. . | $0·6<P<0·7$ | $0·05<P<0·1$ | |
| DD . | M. . | $0·7<P<0·8$ | $0·05<P<0·1$ | $0·8<P<0·9$ |
| 2 weeks after tumour inoculation | | | | |
| SM . | M. . | $0·01<P<0·02$ | | |
| DD . | F. . | $0·6<P<0·7$ | $0·01<P<0·02$ | |
| DD . | M. . | $0·3<P<0·4$ | $0·2<P<0·3$ | $0·4<P<0·5$ |

The authors wish to thank Dr. Ohashi, department of public health for his valuable assistance in the field of statistics, and Dr. Kodama, department of internal medicine, for his advice.

They are also indebted to Yamanouchi Seiyaku Co. Ltd. for their support in conducting this experiment.

This study has been supported by a grant from Ministry of Education, Japanese Government.

### REFERENCES

HEWITT, H. B.—(1953) *Brit. J. Cancer*, **7**, 384.
KAZIWARA, K.—(1954) *Cancer Res.*, **14**, 795.
KODAMA, M.—(1962) *Cancer Res.*, **22**, 1212.
LIEBELT, A. G. AND LIEBELT, R. A.—(1962) *Cancer Res.*, **22**, 1180.
MOORE, G. E., KONDO, T. AND OLIVER, R. J.—(1960) *J. nat. Cancer Inst.*, **25**, 1097.
TAUCHI, H.—(1962) *Ronenbyo*, **6**, 309. (Japanese)
WATSON, B. E. M.—(1958) *J. nat. Cancer Inst.*, **20**, 219.